# Hierarchical analysis of unsuccessful tuberculosis treatment among people living with HIV in Brazil using nationwide surveillance data

**DOI:** 10.1111/hiv.70200

**Published:** 2026-01-28

**Authors:** Lucas Vinícius de Lima, Gabriel Pavinati, Francisco Beraldi‐Magalhães, Rubia Laine de Paula Andrade‐Gonçalves, Aline Aparecida Monroe, Marcela Demitto Furtado, Rosana Rosseto de Oliveira, Daniele Maria Pelissari, Kleydson Bonfim Andrade Alves, Gabriela Tavares Magnabosco

**Affiliations:** ^1^ Universidade Estadual de Maringá Maringá Brazil; ^2^ Fundação de Medicina Tropical Doutor Heitor Vieira Dourado Manaus Brazil; ^3^ Faculdades Pequeno Príncipe Curitiba Brazil; ^4^ Universidade de São Paulo Ribeirão Preto Brazil; ^5^ Ministry of Health Brasília Brazil; ^6^ Pan American Health Organization Brasília Brazil

**Keywords:** cohort studies, coinfection, HIV, protective factors, regression analysis, risk factors, tuberculosis

## Abstract

**Introduction:**

People with tuberculosis‐HIV coinfection face multiple barriers to effective treatment, including social vulnerability, stigma and limited access to healthcare. This study examined factors associated with loss to follow‐up and death among individuals with tuberculosis‐HIV in Brazil.

**Methods:**

We conducted a longitudinal study using a nationally linked database from surveillance systems. Poisson regression models with robust variance were applied to identify factors associated with unfavourable outcomes, guided by a theoretical‐conceptual hierarchical framework.

**Results:**

We analysed data from 54 516 individuals. The median time to loss to treatment follow‐up was 115 days, with a cumulative proportion of 29.56%. Among the most consistent predictors of loss to follow‐up were homelessness (relative risk, RR 1.18; 95% confidence interval, 95% CI 1.16–1.19), tuberculosis retreatment (RR 1.16; 95% CI 1.15–1.17) and drug use (RR 1.15; 95% CI 1.14–1.16), whereas antiretroviral therapy use (RR 0.95; 95% CI 0.95–0.96) showed a negative association. The median time to death during tuberculosis treatment was 27 days, with a cumulative proportion of 27.54%. Higher risk of death was observed among individuals with CD4 counts <350 cells/mm^3^ (RR 1.09; 95% CI 1.08–1.10), those experiencing homelessness (RR 1.08; 95% CI 1.06–1.10) and those with rifampicin resistance (RR 1.11; 95% CI 1.07–1.15).

**Conclusion:**

Key social, clinical and programmatic factors were associated with loss to follow‐up and death during tuberculosis treatment among people with HIV. Addressing these vulnerabilities is essential to improving treatment outcomes and advancing progress towards the 2030 targets.

## INTRODUCTION

Tuberculosis (TB) and human immunodeficiency virus (HIV) remain challenging infections that significantly impact healthcare systems, especially due to their interaction in the progression of one infection over the other [1, 2]. The likelihood of developing TB in people with HIV is about 20 times higher compared with those not infected with the virus [1, 2]. Additionally, individuals with HIV often face unfavourable outcomes in TB treatment, such as death, treatment failure and loss to follow‐up—as shown in previous studies developed worldwide [3, 4].

There is a collaborative effort to end the TB and HIV epidemics by 2030, as established in the third goal of the United Nations (UN) Sustainable Development Goals (SDGs) [5]. In Brazil, between 2010 and 2021, 122 211 new cases of TB‐HIV coinfection were reported, with a trend of decreasing incidence by 4.3% per year (95% confidence interval, 95% CI –5.1 to −3.7) [6]. However, the concomitance of infections disproportionately affects the Brazilian territory, being more notable in areas with high prevalence of HIV and/or incidence of TB [7].

Through the Brazilian Unified Health System, there have been significant advancements in early detection through simultaneous testing for both infections and timely treatment with the availability of antiretroviral therapy combined with the TB therapeutic regimen—especially using directly observed treatment (DOT) [1, 2]. However, people with TB‐HIV still face economic, sociocultural and behavioural barriers to access effective treatment, which arise from factors such as low literacy, poverty, social stigma and fragile access and link to healthcare services [8, 9].

It is important to highlight that the treatment of people with TB‐HIV coinfection requires adherence to two therapies: continuous HIV therapy and TB therapy, which lasts at least six months in typical cases [10]. Despite the complexity, adherence to HIV and TB treatments is crucial for successful outcomes. However, managing care among affected individuals is challenging, especially due to the burden of different therapeutic regimens, poor socioeconomic conditions compounded by illness and the social stigma associated with both infections [11].

Previous studies identified through a state‐of‐the‐art review conducted by the authors reported various factors that interfere with the follow‐up of individuals with TB‐HIV coinfection. The results converged in showing different factors that impacted the treatment of TB‐HIV coinfection, notably the T‐CD4+ lymphocyte count, the use of antiretroviral therapy, socioeconomic status (such as education and race/ethnicity), the individual's age and the presence of comorbidities (such as alcohol, tobacco and illicit drug use).

Despite the range of studies in Brazil, certain perspectives have emerged to address some gaps: the use of an updated database with information on HIV and TB from different systems and the articulation of data related to the individual and their socioeconomic and programmatic context. Therefore, aiming to contribute to the development of more effective policies to achieve the elimination of TB‐HIV, we examined the factors associated with loss to follow‐up and death in individuals with TB‐HIV coinfection in Brazil between 2015 and 2021.

## METHODS

### Design and setting

This is a retrospective cohort study using routinely collected health data from national information systems. Brazil, the study setting, is the largest country in Latin‐America and is composed of 27 federative units organized into five macro‐regions: North, Northeast, South, Southeast and Central‐West. With over 200 million inhabitants in 2021, it has a per capita gross domestic product of BRL 42 247.52 (USD 7712.08), a human development index (HDI) of 0.766 (considered high) and a Gini index of 0.544, representing significant economic inequalities [12, 13].

### Population and period

The population included all people living with HIV who were diagnosed with TB between 2015 and 2021, considering data availability when we accessed data sources. In Brazil, individuals diagnosed with TB‐HIV coinfection are jointly monitored by primary and secondary care services for a minimum period of approximately 180 days—in cases of uncomplicated pulmonary TB—until treatment completion, whether favourable or unfavourable [14]. These follow‐up data are recorded in national‐based information systems.

### Data source

Data were sourced from the Notifiable Diseases Information System (SINAN), Mortality Information System (SIM), Logistic Control System of HIV Medicines (SICLOM) and Laboratory Examination Control System of the National Network for CD4+/CD8+ Lymphocyte Counting and HIV Viral Load (SISCEL). These information systems were linked through probabilistic linkage, as described in the epidemiological bulletin of TB‐HIV coinfection in Brazil [15]. Additionally, data on HDI in 2022 were consulted from the Human Development Atlas—Atlas Brazil [12].

The linkage using Reclink® software occurred in three stages: first between HIV databases (SINAN‐HIV, SIM‐HIV, SISCEL and SICLOM); second between TB databases (SINAN‐TB and SIM‐TB); and third between the results of these two stages. Comparison fields included person's name, mother's name and date of birth; and blocking keys included phonetic codes of the person's first and last names and sex. For control, identification variables were created in each database, allowing search for any variables of interest in the initial databases [15].

### Selection criteria

The inclusion criteria referred to individuals notified in SINAN‐TB with “HIV” or “acquired immunodeficiency syndrome (AIDS)” variables coded as “yes”; and those without these variables filled out, but with records in SINAN‐HIV, SIM‐HIV, SICLOM and/or SISCEL. Initially, we identified 72 089 individuals with HIV who initiated TB treatment at any time between 1 January 2015 and 30 June 2021. Thus, the minimum follow‐up period for all individuals was set at six months, ending on 31 December 2021—totalling an 84‐month cohort.

**FIGURE 1:**
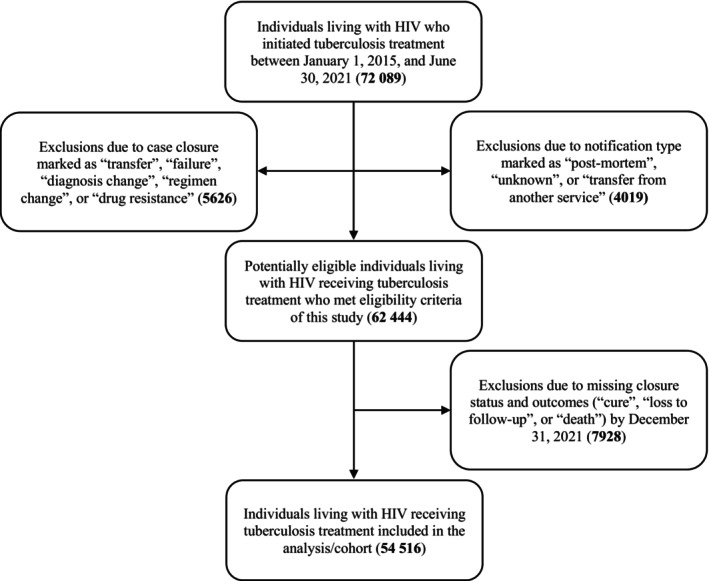
Flow diagram of cohort selection according to the study criteria among people living with HIV undergoing tuberculosis treatment in Brazil, 2015–2021.

A total of 4019 cases in SINAN‐TB with “notification type” marked as “post‐mortem”, “unknown” or “transfer” and of 5626 cases with “closure status” filled out as “transfer”, “diagnosis change”, “regimen change”, “failure” or “resistance” were explicitly excluded due to the absence of follow‐up. Also, 7928 cases lacking “closure status” (i.e., data ignored/in progress) with any of the outcomes—“cure”, “primary abandonment”, “abandonment”, “TB‐related death” and “death from other causes”—until the final date were excluded (Figure 1).

### Variables

The dependent variable comprised three mutually exclusive outcome categories: “cure”, defined as completion of TB treatment with formal discharge from health services; “primary abandonment” and “abandonment”, grouped as “loss to follow‐up”, corresponding to treatment discontinuation before and after 30 days from treatment initiation, respectively, with absence from health services for at least 30 consecutive days; and “TB‐related death” and “death from other causes”, grouped as “death during TB treatment” [14].

According to the operational recommendations of the SINAN, deaths among people living with HIV should not be recorded as “TB‐related death”, even when TB contributes to the fatal outcome; instead, the category “death from other causes” is recommended. However, in routine surveillance practice, inconsistencies are frequently observed, with TB‐HIV cases being recorded as both categories. To minimize differential outcome misclassification, these categories were therefore combined into a single analytical outcome. Regarding the independent variables, we included:

- demographic and socioeconomic: sex (male; female), race/skin colour (self‐declared: white; black; brown; yellow; indigenous), age group (in years: ≤9; 10–19; 20–29; 30–39; 40–49; 50–59; ≥60), macro‐region (North; Southeast; Northeast; South; Central‐West), municipality size (metropolis: >900 000 inhabitants; large: 100 001–900 000 inhabitants; medium: 50 001–100 000 inhabitants; small: ≤50 000 inhabitants), education level (in years: <1; 1–4; 5–8; >8; not applicable), beneficiary of social programmes (no; yes), prison population (no; yes), homeless population (no; yes), immigrant population (no; yes) and HDI of state of residence (very high: 0.800–1.000; high: 0.700–0.799; medium: 0.600–0.699; low: 0.500–0.599; very low: 0.000–0.499);
- epidemiological and clinical: alcohol use (no; yes), drug use (no; yes), tobacco use (no; yes), diabetes (no; yes), mental disorder (no; yes); TB contact evaluation (no; yes), DOT (not; yes), antiretroviral therapy use (not; yes), follow‐up sputum smear examinations (≤2; 3–5; ≥6; not applicable), TB notification type (new case; recurrence; retreatment), clinical form (mixed: pulmonary and extrapulmonary; pulmonary; extrapulmonary), rifampicin resistance in rapid molecular test – RMT (sensitive; resistant; unknown: undetectable/inconclusive; not performed), chest radiography (normal; suggestive of TB; not suggestive; not performed), HIV diagnosis moment (before TB; during TB investigation) and CD4+ T‐lymphocyte count (in cells/mm^3^: ≤350; >350)

These variables are filled out by health professionals—generally nurses or doctors—in the notification and follow‐up form, following guidelines in the manual of recommendations for TB control in Brazil [14]. It should be noted that certain variables may not be completed in information systems (complementary field/not mandatory), and for these situations, a response category indicating absence of data was left. As no progressive association with the “age (in years)” variable was observed, it was analysed categorically by age groups.

Follow‐up time was calculated as the difference between diagnosis date and closure date in SINAN‐TB. Use of antiretroviral therapy during treatment was defined as individuals with dispensation records in SICLOM within 100 days before and 280 days after TB diagnosis. Also, for all TB‐HIV coinfection cases where HIV diagnosis occurred between 100 days before and 280 days after TB diagnosis (180 days of TB treatment plus 100 days after TB treatment completion), we considered that HIV diagnosis occurred due to TB [15].

DOT was defined based on the SINAN field indicating whether the patient effectively received supervised doses according to national programmatic criteria. This variable does not represent eligibility or clinical indication for DOT, but rather the actual execution of the strategy during treatment. Although DOT represents a concrete intervention rather than an indication, it is also influenced by service organization and territorial capacity. Therefore, associations involving DOT should be interpreted as reflecting programmatic performance and access to structured follow‐up.

### Data analysis

Descriptive analysis was performed using absolute frequency (*n*) and relative frequency (%) for nominal and ordinal variables, as well as measures of central tendency and dispersion (median, MD; first quartile, Q1; third quartile, Q3) for discrete variables. Cumulative proportions of loss to follow‐up and death during TB treatment were calculated as the number of individuals experiencing each outcome divided by the sum of cases with that outcome and those cured, and expressed as percentages. 95% CI were estimated using a normal approximation for proportions.

Although time to outcome was described, these intervals reflect the timing of record closure in SINAN rather than the exact date of treatment interruption or death. As closure dates may lag behind the actual occurrence of events and vary according to local operational practices, time‐to‐event information was considered operational rather than epidemiological. For this reason, we deliberately restricted the use of time variables to descriptive analyses and avoided time‐dependent regression models, which could introduce spurious precision and violate model assumptions.

**FIGURE 2:**
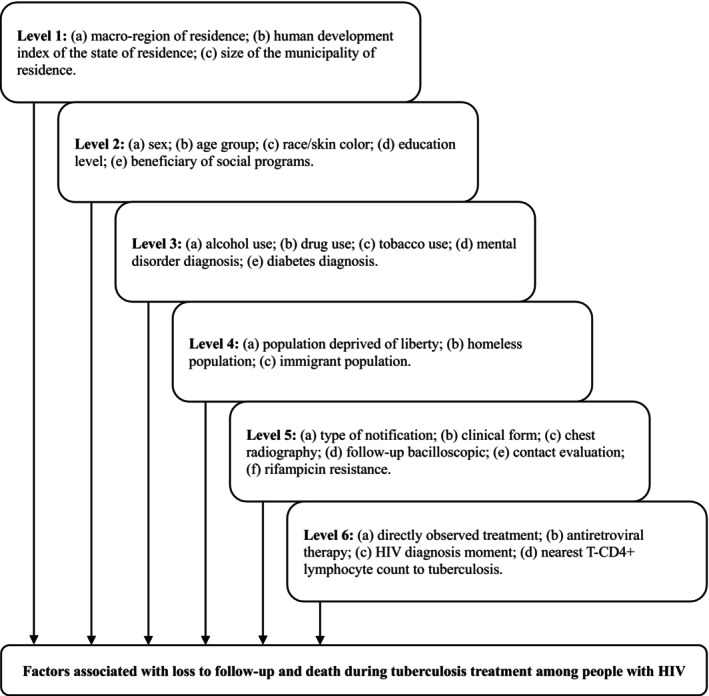
Theoretical‐conceptual model of hierarchical analysis of factors associated with loss to follow‐up and death during tuberculosis treatment among people living with HIV.

Therefore, Poisson regression models with robust variance were used to analyse factors associated with unfavourable outcomes (“cure” versus “loss to follow‐up” and “cure” versus “death during TB treatment”), estimating relative risk (RR) and its 95% CI as measures of association and uncertainty, respectively. Before constructing multiple models, a diagnosis of collinearity of the variables was performed, and we found the absence of multicollinearity. Multiple analyses followed a hierarchical entry, according to the theoretical‐conceptual model developed for this study (Figure 2).

Variables from level 1 were included together, retaining those with a *p*‐value < 0.05 in the Wald test when adjusted against each other (stepwise backward). Subsequently, level 2 variables were included, adjusting them against each other and those remaining from level 1. This process continued up to level 6, adjusting to variables from this level and those remaining from levels 1 to 5. The model underwent validation by epidemiologists researching TB and/or HIV to ensure its coherence. Microsoft Excel® 2016 and IBM SPSS Statistics® (version 25.0) were used for analyses.

Considering that missing information may bias associations by either underestimating or overestimating effect estimates, a post hoc complete‐case analysis was conducted as a sensitivity analysis. Records with at least one missing covariate were excluded to assess whether the magnitude and direction of associations observed in the main models were preserved. Nevertheless, interpretation of findings was primarily based on the main analytical models, which retained missing categories to reflect the structure and completeness of routine surveillance data.

### Ethical aspects

This study followed the Reporting of Studies Conducted using Observational Routinely‐Collected Health Data (RECORD) checklist and respected ethical standards for research involving humans. Although administrative data were used, which are non‐nominal and publicly accessible (upon request via “Fala.BR”; protocol no. 25072.039887/2022–27), this study is part of a doctoral thesis and therefore we obtained ethics approval (opinion no. 6.914.233/2024), following Resolution no. 674/2022, of the National Health Council of Brazil.

## RESULTS

A total of 54 516 people with TB‐HIV coinfection were evaluated. We observed that 55.55% of people with HIV achieved cure of TB treatment in a median time of 207.00 days (Q1 185.00, Q3 269.00), while 23.34% were lost to follow‐up in a median time of 115.00 days (Q1 61.00, Q3 292.80) and 21.11% passed away in a median time of 27.00 days (Q1 8.00, Q3 79.00). The cumulative proportion of loss to follow‐up was 29.56% (95% CI 29.13–29.99), while the cumulative proportion of death during TB treatment was 27.54% (95% CI 27.11–27.97).

The study population was predominantly composed of men, young adults, individuals of mixed race/skin colour and residents of metropolitan areas. A high burden of social and clinical vulnerability was evident, including homelessness, alcohol and drug use, previous TB treatment episodes and limited access to follow‐up examinations. These characteristics reflect structural and programmatic challenges faced by people living with HIV undergoing TB treatment. Detailed distributions of these variables are presented in Table 1.

**TABLE 1:** Description of characteristics of people living with HIV undergoing tuberculosis treatment, according to tuberculosis treatment outcomes, Brazil, 2015–2021.

Variable	Total *N* (%)	Cure *n* (%)	Loss to follow‐up *n* (%)	Death *n* (%)
Sex				
Female	14 665 (26.90)	7582 (25.03)	3935 (30.95)	3148 (27.34)
Male	39 851 (73.10)	22 708 (74.97)	8778 (69.05)	8365 (72.66)
Age group (in years)				
0–9	284 (0.52)	198 (0.65)	34 (0.27)	52 (0.45)
10–19	834 (1.53)	490 (1.62)	229 (1.80)	115 (1.00)
20–29	9884 (18.13)	5331 (17.60)	2937 (23.10)	1616 (14.04)
30–39	17 480 (32.07)	9409 (31.07)	4723 (37.15)	3348 (29.08)
40–49	14 312 (26.25)	7894 (26.06)	3172 (24.95)	3246 (28.19)
50–59	7950 (14.58)	4729 (15.61)	1216 (9.57)	2005 (17.42)
≥60	3538 (6.49)	2110 (6.97)	343 (2.70)	1085 (9.42)
Ignored	234 (0.43)	129 (0.43)	59 (0.46)	46 (0.40)
Race/skin colour				
White	16 204 (29.72)	9591 (31.66)	3331 (26.20)	3282 (28.51)
Black	8312 (15.25)	4313 (14.24)	2401 (18.89)	1598 (13.88)
Brown	25 504 (46.78)	13 973 (46.14)	5994 (47.15)	5537 (48.09)
Yellow	254 (0.47)	159 (0.52)	51 (0.40)	44 (0.38)
Indigenous	169 (0.31)	98 (0.32)	33 (0.26)	38 (0.33)
Ignored	4073 (7.47)	2156 (7.12)	903 (7.10)	1014 (8.81)
Education level (in years)				
<1	1772 (3.25)	983 (3.25)	407 (3.20)	382 (3.32)
1–4	7645 (14.02)	4253 (14.04)	1930 (15.18)	1462 (12.70)
5–8	25 976 (47.65)	15 011 (49.56)	6183 (48.64)	4782 (41.53)
>8	2901 (5.32)	2069 (6.83)	369 (2.90)	463 (4.02)
Not applicable	222 (0.41)	152 (0.50)	29 (0.23)	41 (0.36)
Ignored	16 000 (29.35)	7822 (25.82)	3795 (29.85)	4383 (38.07)
Macro‐region of residence				
South	10 075 (18.48)	5177 (17.09)	2813 (22.13)	2085 (18.11)
Southeast	23 757 (43.58)	13 735 (45.35)	5239 (41.20)	4783 (41.54)
Central‐West	2306 (4.23)	1221 (4.03)	522 (4.11)	563 (4.89)
North	6202 (11.38)	3596 (11.87)	1266 (9.96)	1340 (11.64)
Northeast	12 176 (22.33)	6561 (21.66)	2873 (22.60)	2742 (23.82)
State HDI				
Very high	13 705 (25.14)	8161 (26.94)	2935 (23.09)	2609 (22.66)
High	32 988 (60.51)	17 597 (58.10)	8121 (63.88)	7270 (63.15)
Medium	7823 (14.35)	4532 (14.96)	1657 (13.03)	1634 (14.19)
Municipality size				
Small	7148 (13.11)	4461 (14.73)	1100 (8.65)	1587 (13.78)
Medium	4120 (7.56)	2484 (8.20)	791 (6.22)	845 (7.34)
Large	19 998 (36.68)	11 473 (37.88)	4114 (32.36)	4411 (38.31)
Metropolis	23 250 (42.65)	11 872 (39.19)	6708 (52.77)	4670 (40.57)
Population deprived of liberty				
No	48 155 (88.34)	26 408 (87.18)	11 478 (90.28)	10 269 (89.19)
Yes	3742 (6.86)	2605 (8.60)	691 (5.44)	446 (3.87)
Ignored	2619 (4.80)	1277 (4.22)	544 (4.28)	798 (6.93)
Homeless population				
No	47 232 (86.64)	27 661 (91.32)	9814 (77.20)	9757 (84.75)
Yes	4464 (8.19)	1214 (4.01)	2335 (18.37)	915 (7.95)
Ignored	2820 (5.17)	1415 (4.67)	564 (4.43)	841 (7.30)
Immigrant population				
No	50 415 (92.48)	28 168 (93.00)	11 868 (93.35)	10 379 (90.15)
Yes	345 (0.63)	168 (0.55)	89 (0.70)	88 (0.76)
Ignored	3756 (6.89)	1954 (6.45)	756 (5.95)	1046 (9.09)
Beneficiary of social programme				
No	28 678 (52.61)	16 014 (52.87)	6938 (54.58)	5726 (49.73)
Yes	2748 (5.04)	1640 (5.41)	626 (4.92)	482 (4.19)
Ignored	23 090 (42.35)	12 636 (41.72)	5149 (40.50)	5305 (46.08)
Diabetes				
No	48 926 (89.75)	27 395 (90.44)	11 603 (91.26)	9928 (86.23)
Yes	2078 (3.81)	1289 (4.26)	316 (2.49)	473 (4.11)
Ignored	3512 (6.44)	1606 (5.30)	794 (6.25)	1112 (9.66)
Alcohol use				
No	37 755 (69.25)	22 676 (74.86)	7796 (61.32)	7283 (63.26)
Yes	13 006 (23.86)	5921 (19.55)	4128 (32.47)	2957 (25.68)
Ignored	3755 (6.89)	1693 (5.59)	789 (6.21)	1273 (11.06)
Drug use				
No	37 196 (68.23)	22 915 (75.66)	6714 (52.81)	7567 (65.73)
Yes	12 961 (23.77)	5320 (17.56)	5146 (40.48)	2495 (21.67)
Ignored	4359 (8.00)	2055 (6.78)	853 (6.71)	1451 (12.60)
Tobacco use				
No	36 466 (66.89)	21 458 (70.84)	7597 (59.75)	7411 (64.37)
Yes	13 824 (25.36)	6938 (22.91)	4224 (33.23)	2662 (23.12)
Ignored	4226 (7.75)	1894 (6.25)	892 (7.02)	1440 (12.51)
Mental disorder				
No	49 070 (90.01)	27 733 (91.56)	11 380 (89.51)	9957 (86.48)
Yes	1699 (3.12)	860 (2.84)	489 (3.85)	350 (3.04)
Ignored	3747 (6.87)	1697 (5.60)	844 (6.64)	1206 (10.48)
Type of TB notification				
New case	40 460 (74.22)	24 342 (80.36)	7226 (56.84)	8892 (77.24)
Recurrence	5555 (10.19)	3199 (10.56)	1276 (10.04)	1080 (9.38)
Retreatment	8501 (15.59)	2749 (9.08)	4211 (33.12)	1541 (13.38)
Clinical form of TB				
Pulmonary	40 114 (73.59)	22 273 (73.54)	10 085 (79.33)	7756 (67.37)
Extrapulmonary	9630 (17.66)	5644 (18.63)	1608 (12.65)	2378 (20.65)
Mixed	4772 (8.75)	2373 (7.83)	1020 (8.02)	1379 (11.98)
Chest radiography				
Normal	4108 (7.54)	2487 (8.21)	721 (5.67)	900 (7.82)
Suggestive of TB	38 140 (69.96)	20 992 (69.30)	8866 (69.73)	8282 (71.94)
Not suggestive	950 (1.74)	453 (1.50)	165 (1.30)	332 (2.88)
Not performed	10 565 (19.38)	5951 (19.65)	2781 (21.88)	1833 (15.92)
Ignored	753 (1.38)	407 (1.34)	180 (1.42)	166 (1.44)
Follow‐up sputum smear microscopy				
≤2	33 570 (61.58)	14 234 (47.00)	10 120 (79.60)	9216 (80.05)
3–5	5176 (9.49)	4024 (13.28)	865 (6.80)	287 (2.49)
≥6	8537 (15.66)	7901 (26.08)	466 (3.67)	170 (1.48)
Not applicable	7233 (13.27)	4131 (13.64)	1262 (9.93)	1840 (15.98)
TB contacts evaluation				
No	25 371 (46.54)	12 800 (42.26)	7256 (57.08)	5315 (46.17)
Yes	17 420 (31.95)	13 032 (43.02)	2411 (18.96)	1977 (17.17)
Ignored	11 725 (21.51)	4458 (14.72)	3046 (23.96)	4221 (36.66)
Rifampicin resistance in RMT				
Sensitive	13 026 (23.89)	7444 (24.58)	3662 (28.81)	1920 (16.68)
Resistant	370 (0.68)	149 (0.49)	120 (0.94)	101 (0.88)
Unknown	5528 (10.14)	2954 (9.75)	1290 (10.15)	1284 (11.15)
Not performed	31 635 (58.03)	17 473 (57.69)	6865 (54.00)	7297 (63.38)
Ignored	3957 (7.26)	2270 (7.49)	776 (6.10)	911 (7.91)
Antiretroviral therapy use				
No	27 119 (49.75)	12 467 (41.16)	7402 (58.22)	7250 (62.97)
Yes	27 397 (50.25)	17 823 (58.84)	5311 (41.78)	4263 (37.03)
DOT performed				
No	40 301 (73.93)	20 103 (66.37)	10 678 (83.99)	9520 (82.69)
Yes	14 215 (26.07)	10 187 (33.63)	2035 (16.01)	1993 (17.31)
HIV diagnosis				
Before TB	34 807 (63.85)	18 886 (62.35)	9353 (73.57)	6568 (57.05)
During TB investigation	19 524 (35.81)	11 259 (37.17)	3334 (26.23)	4931 (42.83)
Ignored	185 (0.34)	145 (0.48)	26 (0.20)	14 (0.12)
CD4+ T‐lymphocyte count (in cells/mm^3^)				
≥350	7072 (12.97)	4669 (15.41)	1870 (14.71)	533 (4.63)
<350	19 271 (35.35)	10 778 (35.58)	4427 (34.82)	4066 (35.32)
Ignored	28 173 (51.68)	14 843 (49.01)	6416 (50.47)	6914 (60.05)

Abbreviations: DOT, directly observed treatment; HDI, human development index; HIV, human immunodeficiency virus; RMT, rapid molecular test; TB, tuberculosis.

After hierarchical adjustment, loss to follow‐up was consistently associated with social and programmatic markers. Higher risk was observed among people experiencing homelessness, individuals who use drugs and those undergoing TB retreatment. In contrast, factors related to care organization showed protective associations, including DOT implementation, antiretroviral therapy use, a higher number of follow‐up sputum smear examinations and contact evaluation. Estimates were consistent between the main analytical model and the complete‐case analysis (Table 2).

**TABLE 2:** Follow‐up time, proportion and analysis of factors associated with loss to follow‐up of tuberculosis treatment among people living with HIV, Brazil, 2015–2021.

Variable	Follow‐up time MD (Q1–Q3)^a^	Proportion % (95% CI)	Main model RR (95% CI)	Post hoc model RR (95% CI)
**Level 1**				
Macro‐region of residence				
South	91.00 (22.00–177.00)	35.21 (34.16–36.25)	Reference	Reference
Southeast	125.00 (66.00–202.00)	27.61 (26.98–28.25)	0.94 (0.93–0.95)^b^	0.94 (0.92–0.96)^b^
Central‐West	94.00 (57.25–149.00)	29.95 (27.80–32.10)	0.97 (0.95–0.99)^b^	0.95 (0.91–0.99)^b^
North	120.00 (72.75–184.00)	26.04 (24.81–27.27)	0.92 (0.90–0.93)^b^	0.91 (0.88–0.93)^b^
Northeast	116.00 (62.00–190.00)	30.45 (29.53–31.38)	0.97 (0.96–0.98)^b^	0.96 (0.94–0.99)^b^
State HDI				
Very high	121.00 (66.00–187.00)	26.45 (25.63–27.27)	Reference	Reference
High	111.00 (54.00–191.25)	31.58 (31.01–32.15)	1.02 (1.01–1.03)^b^	1.16 (1.07–1.27)^b^
Medium	119.00 (65.00–188.00)	26.77 (25.67–27.88)	1.00 (0.98–1.02)	1.16 (1.06–1.27)^b^
Municipality size				
Small	122.00 (67.00–208.00)	19.78 (18.73–20.83)	Reference	Reference
Medium	127.00 (74.00–197.00)	24.15 (22.69–25.62)	1.03 (1.01–1.04)^b^	0.97 (0.94–1.01)
Large	116.00 (63.00–185.00)	26.39 (25.70–27.09)	1.05 (1.04–1.06)^b^	1.01 (0.99–1.04)
Metropolis	111.00 (56.00–190.00)	36.10 (35.41–36.79)	1.14 (1.13–1.15)^b^	1.11 (1.08–1.14)^b^
**Level 2**				
Sex				
Female	107.00 (57.00–186.00)	34.71 (33.30–35.03)	Reference	Reference
Male	119.00 (62.00–192.00)	27.88 (27.38–28.37)	0.96 (0.95–0.97)^b^	0.97 (0.95–0.99)^b^
Age group (in years)				
0–9	131.00 (62.00–211.00)	14.66 (10.10–19.21)	Reference	Reference
10–19	129.00 (68.25–198.75)	31.85 (28.44–35.26)	1.21 (1.12–1.31)^b^	1.14 (0.94–1.40)
20–29	116.00 (63.00–190.00)	35.52 (34.49–36.55)	1.26 (1.17–1.36)^b^	1.12 (1.00–1.46)^b^
30–39	112.00 (60.00–189.00)	33.42 (32.64–34.20)	1.24 (1.15–1.34)^b^	1.19 (0.98–1.44)
40–49	118.00 (61.00–190.50)	28.66 (27.82–29.51)	1.19 (1.11–1.29)^b^	1.15 (0.95–1.39)
50–59	113.00 (58.00–189.00)	20.45 (19.43–21.48)	1.12 (1.04–1.21)^b^	1.09 (0.90–1.33)
≥60	114.00 (59.75–182.00)	13.98 (12.61–15.36)	1.06 (0.98–1.15)	1.02 (0.84–1.24)
Race/skin colour				
White	110.00 (52.00–185.00)	25.78 (25.02–26.53)	Reference	Reference
Black	109.00 (51.00–189.00)	35.76 (34.61–36.91)	1.06 (1.05–1.07)^b^	1.05 (1.02–1.08)^b^
Brown	119.00 (63.00–192.00)	30.02 (29.38–30.66)	1.04 (1.03–1.05)^b^	1.03 (1.00–1.05)^b^
Yellow	117.00 (48.75–197.25)	24.29 (18.49–30.09)	0.99 (0.94–1.04)	0.96 (0.87–1.06)
Indigenous	155.50 (87.75–247.50)	25.19 (17.76–32.62)	1.02 (0.96–1.08)	0.93 (0.84–1.02)
Education level (in years)				
<1	122.00 (64.00–210.00)	29.28 (26.89–31.67)	Reference	Reference
1–4	115.00 (61.00–192.00)	31.21 (30.60–32.37)	0.99 (0.97–1.01)	0.99 (0.95–1.03)
5–8	119.00 (62.00–191.00)	29.17 (28.56–29.79)	0.96 (0.94–0.97)^b^	0.95 (0.92–0.99)^b^
>8	107.50 (62.00–188.75)	15.14 (13.71–16.56)	0.86 (0.84–0.88)^b^	0.83 (0.79–0.87)^b^
Not applicable	140.00 (62.00–212.00)	16.02 (10.68–21.37)	1.06 (0.97–1.16)	0.94 (0.77–1.15)
Beneficiary of social programme				
No	110.00 (55.00–186.00)	30.23 (29.63–30.82)	Reference	Reference
Yes	127.00 (66.75–196.00)	27.63 (25.78–29.47)	0.98 (0.96–0.99)^b^	0.98 (0.95–1.01)
**Level 3**				
Alcohol use				
No	117.00 (61.00–189.00)	25.58 (25.09–26.07)	Reference	Reference
Yes	112.00 (61.00–188.00)	41.08 (40.12–42.04)	1.04 (1.03–1.05)^b^	1.04 (1.02–1.06)^b^
Drug use				
No	120.00 (62.00–194.00)	22.66 (22.18–23.14)	Reference	Reference
Yes	108.00 (59.00–182.00)	49.17 (48.21–50.13)	1.15 (1.14–1.16)^b^	1.15 (1.12–1.17)^b^
Diabetes				
No	115.00 (61.00–189.00)	29.75 (29.30–30.21)	Reference	Reference
Yes	119.00 (61.00–183.50)	19.96 (17.74–21.63)	0.96 (0.94–0.97)^b^	1.01 (0.97–1.06)
**Level 4**				
Population deprived of liberty				
No	114.00 (61.00–189.00)	30.30 (29.83–30.76)	Reference	Reference
Yes	122.00 (68.25–194.75)	20.96 (19.58–22.35)	0.92 (0.91–0.93)^b^	0.92 (0.89–0.95)^b^
Homeless population				
No	120.00 (63.00–192.00)	26.19 (25.74–26.63)	Reference	Reference
Yes	93.00 (39.00–170.00)	65.79 (64.23–67.35)	1.18 (1.16–1.19)^b^	1.19 (1.16–1.23)^b^
**Level 5**				
Type of TB notification				
New case	119.00 (62.00–186.00)	22.89 (22.43–22.35)	Reference	Reference
Recurrence	118.00 (61.00–183.00)	28.51 (27.19–29.87)	1.03 (1.02–1.04)^b^	1.03 (1.01–1.06)^b^
Retreatment	109.00 (56.00–202.00)	60.50 (59.35–61.65)	1.16 (1.15–1.17)^b^	1.17 (1.15–1.20)^b^
Clinical form of TB				
Pulmonary	114.00 (61.00–188.00)	31.17 (30.66–31.67)	Reference	Reference
Extrapulmonary	122.00 (62.00–198.00)	22.17 (21.22–23.13)	0.93 (0.92–0.94)^b^	0.96 (0.91–1.02)
Mixed	117.00 (61.00–194.25)	30.06 (28.52–31.60)	0.95 (0.94–0.96)^b^	0.96 (0.94–0.99)^b^
Chest radiography				
Normal	115.50 (60.25–195.00)	22.48 (21.03–23.92)	Reference	Reference
Suggestive of TB	113.00 (60.00–188.00)	29.69 (29.18–30.21)	1.01 (1.00–1.03)^b^	1.02 (0.99–1.05)
Not suggestive	97.00 (34.00–182.00)	26.70 (23.21–30.19)	1.01 (0.98–1.04)	1.02 (0.96–1.10)
Not performed	121.00 (64.00–196.00)	31.85 (30.87–32.83)	1.03 (1.02–1.05)^b^	1.03 (1.00–1.06)^b^
Follow‐up sputum smear microscopy				
≤2	104.00 (58.00–181.00)	41.55 (40.93–42.17)	Reference	Reference
3–5	163.00 (120.00–225.00)	17.96 (16.62–18.76)	0.86 (0.85–0.87)^b^	0.86 (0.84–0.88)^b^
≥6	189.00 (139.25–240.75)	5.57 (5.08–6.06)	0.79 (0.79–0.80)^b^	0.82 (0.80–0.83)^b^
Not applicable	122.00 (60.00–201.00)	23.40 (22.27–24.53)	0.96 (0.95–0.98)^b^	0.95 (0.90–1.01)
TB contacts evaluation				
No	108.00 (59.00–183.00)	36.18 (35.51–36.84)	Reference	Reference
Yes	150.00 (91.00–217.00)	15.61 (15.04–16.18)	0.92 (0.91–0.93)^b^	0.91 (0.89–0.92)^b^
Rifampicin resistance in RMT				
Sensitive	120.00 (63.00–189.00)	32.97 (32.10–33.85)	Reference	Reference
Resistant	119.00 (54.75–234.25)	44.61 (38.67–50.55)	1.05 (1.01–1.09)^b^	0.98 (0.89–1.08)
Unknown	112.00 (60.00–191.00)	30.40 (29.01–31.78)	0.97 (0.96–0.98)^b^	0.98 (0.95–1.01)
Not performed	112.00 (60.00–190.00)	28.21 (27.64–28.77)	0.98 (0.97–0.98)^b^	0.98 (0.96–0.99)^b^
**Level 6**				
DOT performed				
No	111.00 (60.00–185.00)	34.69 (34.16–35.22)	Reference	Reference
Yes	138.00 (75.00–217.00)	16.65 (15.99–17.31)	0.92 (0.92–0.93)^b^	0.94 (0.92–0.95)^b^
Antiretroviral therapy use				
No	105.00 (26.00–182.00)	37.25 (36.58–37.93)	Reference	Reference
Yes	127.00 (67.00–202.00)	22.96 (22.42–23.50)	0.95 (0.95–0.96)^b^	0.94 (0.92–0.96)^b^
HIV diagnosis				
Before TB	113.00 (60.00–188.00)	33.12 (32.57–33.67)	Reference	Reference
During TB investigation	120.00 (62.00–196.00)	22.85 (22.17–23.53)	0.96 (0.95–0.96)^b^	0.96 (0.94–0.97)^b^

*Note*: (1) only covariates retained in the final multivariable model after hierarchical adjustment at each level and, when applicable, adjustment for preceding levels are shown. (2) relative risks close to 1.00 indicate modest relative differences and should be interpreted in light of population‐level effects and the large sample size. (3) the post hoc complete‐case analysis included 6764 individuals with “cure” outcome and 301 individuals with “loss to follow‐up” outcome.

Abbreviations: 95% CI, 95% confidence interval (lower limit–upper limit); DOT, directly observed treatment; HDI, human development index; HIV, human immunodeficiency virus; MD, median; Q1–Q3, first quartile–third quartile; RMT, rapid molecular test; RR, relative risk; TB, tuberculosis.

^a^ Excluding cases with missing time.

^b^ *p*‐value < 0.05 in the Wald test.

Death during TB treatment was strongly associated with clinical and social vulnerabilities. Higher risk was observed among individuals with CD4 counts below 350 cells/mm^3^, users of alcohol, tobacco and drugs, people experiencing homelessness, and those undergoing retreatment. Conversely, antiretroviral therapy use, performance of follow‐up examinations, and contact evaluation were associated with a lower risk of death. Although effect sizes were modest, associations were consistent and showed similar direction in the sensitivity analysis (Table 3).

**TABLE 3:** Follow‐up time, proportion and analysis of factors associated with death during tuberculosis treatment among people living with HIV, Brazil, 2015–2021.

Variable	Follow‐up time MD (Q1–Q3)^a^	Proportion % (95% CI)	Main model RR (95% CI)	Post hoc model RR (95% CI)
**Level 1**				
Macro‐region of residence				
South	24.00 (7.00–74.00)	28.71 (27.67–29.75)	Reference	Reference
Southeast	28.00 (8.00–83.00)	25.83 (25.20–26.46)	0.98 (0.97–1.00)^b^	0.94 (0.92–0.96)^b^
Central‐West	31.00 (10.00–81.50)	31.56 (29.40–33.71)	1.02 (1.00–1.04)^b^	0.98 (0.94–1.03)
North	24.00 (7.00–75.00)	27.15 (25.91–28.39)	0.99 (0.98–1.01)	0.90 (0.88–0.93)^b^
Northeast	28.00 (9.00–77.00)	29.47 (28.55–30.40)	1.02 (1.00–1.03)^b^	0.97 (0.94–0.99)^b^
State HDI				
Very high	27.00 (7.00–81.00)	24.22 (23.42–25.03)	Reference	Reference
High	27.00 (8.00–78.00)	29.24 (28.67–29.80)	1.03 (1.02–1.04)^b^	1.14 (1.06–1.22)^b^
Medium	28.00 (8.00–78.00)	26.50 (25.40–27.60)	1.00 (0.98–1.01)	1.13 (1.05–1.22)^b^
Municipality size				
Small	28.50 (8.00–78.00)	26.24 (25.13–27.35)	Reference	Reference
Medium	31.00 (8.00–75.50)	25.38 (23.90–26.86)	0.99 (0.98–1.00)	0.97 (0.94–1.00)
Large	26.00 (8.00–77.00)	27.77 (27.07–28.47)	1.01 (1.00–1.02)^b^	0.98 (0.95–1.00)
Metropolis	26.50 (8.00–81.25)	28.23 (27.55–28.92)	1.02 (1.01–1.03)^b^	1.01 (0.98–1.03)
**Level 2**				
Sex				
Female	27.00 (7.00–84.00)	29.34 (28.48–30.20)	Reference	Reference
Male	27.00 (8.00–77.00)	26.92 (26.43–27.41)	0.96 (0.95–0.97)^b^	0.99 (0.97–1.01)
Age group (in years)				
0–9	23.00 (6.00–96.00)	20.80 (15.77–25.83)	Reference	Reference
10–19	21.00 (5.00–106.00)	19.01 (15.88–22.13)	1.01 (0.92–1.10)	1.05 (0.99–1.11)
20–29	28.00 (8.00–86.00)	23.26 (22.27–24.26)	1.05 (0.96–1.14)	1.10 (1.06–1.14)^b^
30–39	28.00 (8.00–84.00)	26.24 (25.48–27.01)	1.07 (0.98–1.17)	1.10 (1.07–1.14)^b^
40–49	27.00 (8.00–78.00)	29.14 (28.29–29.98)	1.09 (1.00–1.19)^b^	1.13 (1.10–1.17)^b^
50–59	25.00 (8.00–74.00)	29.77 (28.68–30.87)	1.10 (1.01–1.20)^b^	1.15 (1.12–1.19)^b^
≥60	27.00 (9.00–72.00)	33.96 (32.32–35.60)	1.13 (1.04–1.24)^b^	1.21 (1.16–1.27)^b^
Race/skin colour				
White	26.50 (7.00–76.75)	25.50 (24.74–26.25)	Reference	Reference
Black	33.00 (9.00–90.00)	27.03 (25.90–28.17)	1.00 (0.99–1.01)	1.02 (1.00–1.05)^b^
Brown	26.00 (8.00–77.00)	28.38 (27.75–29.01)	1.01 (1.00–1.02)^b^	1.01 (0.99–1.03)
Yellow	55.00 (24.00–130.50)	21.67 (16.01–27.34)	0.96 (0.91–1.00)	0.96 (0.87–1.05)
Indigenous	26.00 (7.75–105.25)	27.94 (20.40–35.48)	1.02 (0.96–1.08)	0.91 (0.85–0.98)^b^
Education level (in years)				
<1	35.00 (10.00–104.00)	27.99 (25.60–30.37)	Reference	Reference
1–4	37.00 (11.00–95.00)	25.58 (24.45–26.71)	0.98 (0.96–1.00)	0.98 (0.94–1.03)
5–8	29.00 (8.00–84.00)	24.16 (23.56–24.76)	0.98 (0.96–1.00)	0.99 (0.95–1.03)
>8	28.00 (8.00–74.00)	18.29 (16.78–19.79)	0.94 (0.92–0.96)^b^	0.97 (0.92–1.02)
Not applicable	25.00 (6.50–80.00)	21.24 (15.47–27.01)	1.03 (0.93–1.14)	1.07 (0.97–1.18)
Beneficiary of social programme				
No	27.00 (8.00–79.00)	26.34 (25.75–26.92)	Reference	Reference
Yes	43.50 (12.75–114.25)	22.71 (20.93–24.50)	0,97 (0,95–0,98)^b^	0.97 (0.94–1.00)^b^
**Level 3**				
Alcohol use				
No	28.00 (8.00–81.00)	24.31 (23.82–24.80)	Reference	Reference
Yes	29.00 (8.00–79.00)	33.31 (32.33–34.29)	1.06 (1.05–1.07)^b^	1.02 (1.00–1.05)^b^
Drug use				
No	28.00 (8.00–79.00)	24.82 (24.34–25.31)	Reference	Reference
Yes	30.00 (8.00–87.25)	31.93 (30.89–32.96)	1.04 (1.03–1.05)^b^	1.04 (1.02–1.07)^b^
Tobacco use				
No	27.00 (8.00–80.00)	25.67 (25.17–26.18)	Reference	Reference
Yes	31.00 (9.00–85.00)	27.73 (26.83–28.62)	0.97 (0.96–0.98)^b^	0.98 (0.96–1.00)^b^
Diabetes				
No	28.00 (8.00–80.00)	26.60 (26.15–27.05)	Reference	Reference
Yes	25.50 (7.00–75.25)	26.84 (24.78–28.91)	0.97 (0.95–0.99)^b^	1.02 (0.97–1.07)
**Level 4**				
Population deprived of liberty				
No	27.00 (8.00–78.00)	28.00 (27.54–28.46)	Reference	Reference
Yes	33.00 (7.00–120.50)	14.62 (13.36–15.87)	0.89 (0.88–0.90)^b^	0.94 (0.91–0.97)^b^
Homeless population				
No	27.00 (8.00–79.75)	26.08 (25.63–26.52)	Reference	Reference
Yes	27.00 (7.00–78.25)	42.98 (40.88–45.08)	1.08 (1.06–1.10)^b^	1.08 (1.03–1.13)^b^
Immigrant population				
No	27.00 (8.00–19.00)	26.93 (26.48–27.37)	Reference	Reference
Yes	33.00 (6.00–108.00)	34.38 (28.56–40.19)	1.06 (1.01–1.11)^b^	0.92 (0.85–0.99)^b^
**Level 5**				
Type of TB notification				
New case	25.00 (8.00–71.00)	26.76 (26.28–27.23)	Reference	Reference
Recurrence	30.50 (9.00–98.75)	25.24 (23.94–26.54)	0.98 (0.97–0.99)^b^	0.98 (0.96–1.01)
Retreatment	42.00 (10.00–128.00)	35.92 (34.49–37.36)	1.03 (1.02–1.04)^b^	1.08 (1.05–1.11)^b^
Clinical form of TB				
Pulmonary	26.00 (7.00–81.00)	25.83 (25.33–26.32)	Reference	Reference
Extrapulmonary	31.00 (10.00–81.00)	29.64 (28.64–30.64)	0.97 (0.95–0.98)^b^	1.02 (0.96–1.08)
Mixed	24.00 (7.00–70.00)	36.75 (35.21–38.30)	1.02 (1.01–1.03)^b^	1.02 (0.99–1.05)
Chest radiography				
Normal	34.00 (11.00–89.00)	26.57 (25.08–28.06)	Reference	Reference
Suggestive of TB	25.00 (7.00–75.00)	28.29 (27.78–28.81)	1.02 (1.01–1.03)^b^	1.01 (0.98–1.04)
Not suggestive	19.00 (6.00–64.00)	42.29 (38.84–45.75)	1.08 (1.05–1.11)^b^	1.03 (0.96–1.11)
Not performed	33.00 (9.00–97.00)	23.55 (22.61–24.49)	1.00 (0.99–1.01)	0.98 (0.95–1.02)
Follow‐up sputum smear microscopy				
≤2	24.00 (7.00–70.00)	39.30 (38.68–39.93)	Reference	Reference
3–5	119.00 (69.25–175.50)	6.66 (5.91–7.40)	0.80 (0.79–0.80)^b^	0.86 (0.85–0.88)^b^
≥6	167.00 (114.00–230.50)	2.11 (1.79–2.42)	0.77 (0.77–0.78)^b^	0.84 (0.83–0.85)^b^
Not applicable	31.00 (10.00–80.00)	30.82 (29.64–31.99)	0.97 (0.96–0.99)^b^	0.93 (0.88–0.99)^b^
TB contacts evaluation				
No	25.00 (8.00–76.00)	29.34 (28.68–30.00)	Reference	Reference
Yes	51.00 (17.00–123.00)	13.17 (12.63–13.71)	0.93 (0.92–0.94)^b^	0.92 (0.91–0.94)^b^
Rifampicin resistance in RMT				
Sensitive	30.00 (8.00–89.50)	20.50 (19.96–21.32)	Reference	Reference
Resistant	24.00 (9.00–74.00)	40.40 (34.32–46.48)	1.11 (1.07–1.15)^b^	1.10 (1.01–1.20)^b^
Unknown	31.00 (11.00–86.00)	30.30 (28.91–31.68)	1.02 (1.00–1.03)^b^	1.02 (0.99–1.05)
Not performed	25.00 (7.00–75.50)	29.46 (28.89–30.03)	1.03 (1.02–1.04)^b^	1.01 (0.99–1.03)
**Level 6**				
DOT performed				
No	26.00 (7.00–75.00)	32.14 (31.61–32.67)	Reference	Reference
Yes	35.00 (11.00–98.00)	16.36 (15.71–17.02)	0.97 (0.96–0.97)^b^	1.00 (0.99–1.02)
Antiretroviral therapy use				
No	21.00 (7.00–63.00)	36.77 (36.10–37.44)	Reference	Reference
Yes	43.00 (12.00–105.00)	19.30 (18.78–19.82)	0.87 (0.87–0.88)^b^	0.84 (0.82–0.86)^b^
HIV diagnosis				
Before TB	29.00 (8.00–91.00)	25.80 (25.27–26.34)	Reference	Reference
During TB investigation	24.00 (8.00–67.00)	30.46 (29.75–31.17)	1.04 (1.04–1.05)^b^	1.00 (0.98–1.01)
CD4+ T‐lymphocyte count (in cells/mm^3^)				
≥350	34.00 (11.00–104.00)	10.25 (9.42–11.07)	Reference	Reference
<350	34.00 (10.00–94.00)	27.39 (26.67–28.11)	1.09 (1.08–1.10)^b^	1.10 (1.08–1.11)^b^

*Note*: (1) only covariates retained in the final multivariable model after hierarchical adjustment at each level and, when applicable, adjustment for preceding levels are shown. (2) relative risks close to 1.00 indicate modest relative differences and should be interpreted in light of population‐level effects and the large sample size. (3) the post hoc complete‐case analysis included 5256 individuals with “cure” outcome and 959 individuals with “death during tuberculosis treatment” outcome.

Abbreviations: 95% CI, 95% confidence interval (lower limit–upper limit); DOT, directly observed treatment; HDI, human development index; HIV, human immunodeficiency virus; MD, median; Q1–Q3, first quartile–third quartile; RMT, rapid molecular test; RR, relative risk; TB, tuberculosis.

^a^ Excluding cases with missing time.

^b^ *p*‐value < 0.05 in the Wald test.

## DISCUSSION

This national retrospective cohort study with 54 516 people with TB‐HIV coinfection demonstrated that the treatment of these infections is complex and surrounded by various factors that lead to loss of follow‐up and death. This indicates the need to recognize the coinfection as a syndemic: the syndemic model focuses on the biosocial complex, which consists of interactive, coexisting or sequential diseases, and the social and environmental factors that promote and amplify the negative effects of the interaction of these diseases [16, 17].

Historically, the southern region of Brazil has had the highest burden of HIV due to several complex factors [18]. Although HIV diagnosis and treatment indicators are better in the South compared with the Southeast, Northeast, Central‐West and North [19], the proportions of loss to follow‐up and death due to TB‐HIV were lower in these regions. These differences may partly reflect regional variation in the quality of surveillance and reporting practices within SINAN [20], which can influence patterns of TB detection and notification.

In Brazil, men constitute the group most affected by HIV and TB [6]. Although higher proportions of antiretroviral therapy follow‐up among men were observed—both in national indicators [19] and in our study—these differences should be interpreted cautiously. They may reflect differential patterns of service utilization, engagement in care or reporting practices rather than individual behavioural choices. The mechanisms underlying sex differences in treatment outcomes remain insufficiently explored and warrant further investigation.

People with higher educational levels—which can facilitate access to health resources—tend to have greater access to higher income and better understanding of the importance of continuous treatment [21]. These factors are commonly associated with higher health literacy and are frequently observed among individuals with greater continuity of treatment and engagement with health services. This underscores the relevance of health education and professional guidance in care settings where greater treatment continuity and service engagement are observed.

Regarding vulnerable groups, the incarcerated population showed a lower probability of experiencing unfavourable outcomes of TB‐HIV. Although the prison environment is more conducive to the transmission of TB, those undergoing treatment tend to receive closer monitoring from healthcare professionals, which may contribute to lower loss to follow‐up and mortality [3]. In Brazil, prison healthcare teams have been crucial in providing access to and ensuring healthcare for this population—often neglected and rendered invisible by public policies [22].

In this context, the quality of healthcare services is closely linked to successful outcomes for coinfection. Individuals undergoing treatment who had monthly bacilloscopic tests, whose contacts were evaluated by healthcare professionals and who lived in metropolises, which may offer greater availability of health services and diagnostic resources, showed a lower probability of loss to follow‐up or death due to TB‐HIV. Improving the work processes and infrastructure of primary healthcare has the potential to enhance the diagnosis, monitoring and treatment of TB [23].

We must highlight that certain strategies and policies are crucial for improving TB treatment outcomes. The implementation of DOT and the use of antiretroviral therapy have been associated with reductions in loss to follow‐up and death due to coinfection [24]. Antiretroviral therapy use was strongly associated with a lower risk of death and lower CD4+ T‐cell counts were also associated with mortality. These findings highlight the importance of timely HIV treatment as recommended public policies, although causal inferences should be avoided.

Dual testing—that is, offering HIV tests to people undergoing TB treatment or regular TB testing for people living with HIV—has also been important in reducing loss to follow‐up. In Brazil, this constitutes a strategy in primary healthcare and specialized care services. The discovery of HIV through TB screening, often at basic health units, highlights the potential for integration between different levels of care. Despite this, the reality remains different: there is a binary approach rather than an interactive and shared management perspective of care [25].

We should mention that the clinical form of TB can also influence treatment outcomes. More complex forms (extrapulmonary and mixed) were significantly associated with reduced loss to follow‐up in a retrospective cohort in Brazil [26]. The authors suggested that these results might be related to the care model, where such individuals must also be attended to at outpatient and/or hospital levels [26]. This could contribute, for example, to reducing the chances of treatment discontinuation, primarily by involving more teams and professionals in the care process.

Individuals undergoing TB retreatment showed a higher likelihood of subsequent loss to follow‐up. This association may reflect cumulative social vulnerability, previous barriers to care or challenges in treatment continuity rather than individual choice. Given that these results are consistent [3, 26], current strategies aimed at improving treatment continuity and adherence may require reassessment to increase public awareness. This situation could, for example, lead to multidrug‐resistant TB, which was associated with a higher risk of death in our study.

Other behavioural factors, such as alcohol and drug use, were also associated with unfavourable treatment outcomes in our study. These associations may reflect challenges in treatment continuity, social vulnerability and greater clinical complexity, although the underlying mechanisms cannot be directly assessed in registry‐based analyses [27, 28]. Such vulnerabilities may have greater implications among older individuals, who are at increased risk of clinical deterioration, including hospitalization and death during treatment.

The social factor also influenced the treatment for TB‐HIV coinfection. We observed that homeless individuals in Brazil had higher probabilities of not completing treatment for the dual infection. An ecological study concluded that most Brazilian federative units had insufficient results, particularly noting low HIV testing rates, high rates of coinfection and minimal implementation of DOT [29]. Therefore, there is an urgent need to improve and implement new strategies for the healthcare of the homeless population.

The findings of our study present an alarming scenario for Brazil: high proportions of loss to follow‐up and death due to TB‐HIV coinfection. Pavinati and colleagues, by analysing the temporal trend of TB cure indicators in Brazil, identified an annual reduction of 1.11% (95% CI −1.42 to −0.85) in the cure rate for individuals with TB‐HIV coinfection; this decreasing trend was observed in 70.37% of the country's federative units [30]. Furthermore, the authors reported that only 48.68% of coinfection cases were cured in Brazil between 2001 and 2022 [30].

Despite the potential contribution of our results, we must point out some limitations. These weaknesses mainly stem from the use of secondary data, which are subject to underreporting, incompleteness, inconsistency and under‐detection. The probabilistic linkage between databases was performed by the Brazilian Ministry of Health prior to data availability to the authors, as part of routine surveillance procedures. We therefore rely on previously published technical reports describing the linkage methodology and acknowledge potential linkage error as a study limitation.

This study is also subject to potential time‐related bias. Antiretroviral use was defined based on dispensing records within a predefined window around TB diagnosis, while DOT and follow‐up examinations are processes recorded during treatment. Consequently, individuals must remain alive and engaged in care to be classified as exposed to these interventions, which may partially explain the observed protective associations. These findings should therefore be interpreted as reflecting continuity of care and programmatic performance rather than direct causal effects.

Furthermore, it is important to note that the completion date considered in this study as the end of TB treatment among people living with HIV corresponds to an operational date recorded in SINAN. Consequently, treatment interruption or death may have occurred earlier than the date registered in the system. Also, as outcome closure in SINAN depends on routine service reporting and administrative practices, the observed associations are more likely to reflect differences in programmatic performance and continuity of care than individual clinical trajectories.

Analyses that include missing categories or rely on complete‐case approaches may introduce bias. Although multiple imputation was not performed to address ignored variables, this decision was based on the non‐random nature of missingness in routine surveillance data. Nevertheless, individuals excluded from the complete‐case analysis likely differed systematically from those retained, particularly regarding access to laboratory testing and completeness of socioeconomic information, which may affect the interpretation and generalizability of the findings.

All in all, our study highlighted the complexity of demographic, socioeconomic, epidemiological and clinical factors that interfere with the follow‐up of people undergoing treatment for TB‐HIV coinfection in Brazil. We emphasized that different spheres need to be considered in the context of the lives of those affected, based on the recognition of infections as a complex syndemic. Therefore, it is necessary to address all the factors that, in some way, hinder follow‐up to achieve better results in the quest for the elimination of TB‐HIV by 2030.

## Data Availability

The data that support the findings of this study are available from the corresponding author upon reasonable request.
